# The TiHoCL panel for canine lymphoma: a feasibility study integrating functional genomics and network biology approaches for comparative oncology targeted NGS panel design

**DOI:** 10.3389/fvets.2023.1301536

**Published:** 2023-12-08

**Authors:** Silvia Fibi-Smetana, Camila Inglis, Daniela Schuster, Nina Eberle, José Luis Granados-Soler, Wen Liu, Saskia Krohn, Christian Junghanss, Ingo Nolte, Leila Taher, Hugo Murua Escobar

**Affiliations:** ^1^Institute of Biomedical Informatics, Graz University of Technology, Graz, Austria; ^2^Small Animal Clinic, University of Veterinary Medicine Hannover Foundation, Hannover, Germany; ^3^Clinic for Hematology, Oncology and Palliative Care, Rostock University Medical Center, University of Rostock, Rostock, Germany; ^4^Division of Bioinformatics, Department of Biology, Friedrich-Alexander-University, Erlangen, Germany; ^5^Institute for Biostatistics and Informatics in Medicine and Ageing Research, Rostock University Medical Center, University of Rostock, Rostock, Germany; ^6^UQVETS Small Animal Hospital, School of Veterinary Science, The University of Queensland, Gatton, QLD, Australia

**Keywords:** targeted next-generation sequencing, canine lymphoma, genomic variants, comparative oncology, personalized oncology, patient stratification, comparative genomics, network biology

## Abstract

Targeted next-generation sequencing (NGS) enables the identification of genomic variants in cancer patients with high sensitivity at relatively low costs, and has thus opened the era to personalized human oncology. Veterinary medicine tends to adopt new technologies at a slower pace compared to human medicine due to lower funding, nonetheless it embraces technological advancements over time. Hence, it is reasonable to assume that targeted NGS will be incorporated into routine veterinary practice in the foreseeable future. Many animal diseases have well-researched human counterparts and hence, insights gained from the latter might, in principle, be harnessed to elucidate the former. Here, we present the TiHoCL targeted NGS panel as a proof of concept, exemplifying how functional genomics and network approaches can be effectively used to leverage the wealth of information available for human diseases in the development of targeted sequencing panels for veterinary medicine. Specifically, the TiHoCL targeted NGS panel is a molecular tool for characterizing and stratifying canine lymphoma (CL) patients designed based on human non-Hodgkin lymphoma (NHL) research outputs. While various single nucleotide polymorphisms (SNPs) have been associated with high risk of developing NHL, poor prognosis and resistance to treatment in NHL patients, little is known about the genetics of CL. Thus, the ~100 SNPs featured in the TiHoCL targeted NGS panel were selected using functional genomics and network approaches following a literature and database search that shielded ~500 SNPs associated with, in nearly all cases, human hematologic malignancies. The TiHoCL targeted NGS panel underwent technical validation and preliminary functional assessment by sequencing DNA samples isolated from blood of 29 lymphoma dogs using an Ion Torrent^™^ PGM System achieving good sequencing run metrics. Our design framework holds new possibilities for the design of similar molecular tools applied to other diseases for which limited knowledge is available and will improve drug target discovery and patient care.

## 1 Introduction

Driven by plummeting costs, next-generation sequencing (NGS) has revolutionized biomedical research, playing an instrumental role in advancing our understanding of the molecular basis of various diseases (1). NGS has numerous applications, ranging from whole-genome (re)sequencing to targeted sequencing for variant identification or confirmation. In contrast to whole-genome sequencing, targeted NGS focuses on a specific set of genomic loci that are likely to be involved in the phenotype of interest, delivering higher coverage levels at a more affordable cost, and making it amenable to samples containing small DNA amounts. Furthermore, targeted NGS produces substantially smaller datasets, which are easier to manage and analyze (2). These attributes make targeted NGS particularly well suited to detect and characterize specific tumor cell sub-populations, such as subclones harboring drug resistant variants (3), and hence, very attractive for clinical oncology (4, 5). Indeed, in human medicine, targeted NGS has become a common tool to diagnose and monitor cancer, as well as to select therapeutic agents and quantify treatment resistance.

Due to various factors, including financial considerations and the diversity of animal species and breeds, veterinary medicine tends to adopt technological advances more slowly than human medicine. Nonetheless, there are a number of cases in which the use of targeted NGS has already proved to be cost-effective. For example, targeted NGS panels are routinely used in the clinic to detect ovine and equine pathogens (6, 7). And given the growing significance of the well-being of companion animals in our society, it is inevitable that cutting-edge technologies such as targeted NGS will be progressively adopted to improve their health and quality of life.

Targeted NGS requires a certain knowledge of the genetic basis of the disease of interest, in particular, of the variants or mutations that are associated with it. This can pose challenges for diseases that lack extensive research, as it is generally the case for animal diseases. However, biological processes and genetic mechanisms are frequently conserved across species, making findings in human medicine relevant to veterinary medicine –and vice versa–, and facilitating research and technology transfer between the two fields (8). Consistently, veterinary medicine often resorts to comparative approaches to gain insights into health and disease. Herein, we illustrate how knowledge can be leveraged across species and/or diseases to develop a targeted sequencing panel for canine lymphoma (CL).

CL is a spontaneous disease that closely resembles human Non-Hodgkin lymphoma (NHL), a heterogeneous group of lymphoid malignancies, with different cells of origin and biological behaviors. Specifically, CL and NHL present similar clinical, histological, cytogenetic, and molecular features (9, 10). Furthermore, CL and NHL are classified according to analogous histologic systems and treated with the same chemotherapeutic agents. In particular, the standard backbone treatment for CL and NHL is a chemotherapy combination known as CHOP [cyclophosphamide, hydroxydaunorubicin, oncovin/vincristine, prednisone or prednisolone; (11, 12)]. Groundbreaking CD20 antibody strategies, as used in humans, are unfortunately not available in dogs, thus conventional chemotherapy remains the key strategy for CL. Naturally, although CL and NHL are similar in many regards, they also exhibit important differences. Most notably, while NHL patients often respond well to treatment and over 50% of them are cured after initial therapy (9, 10, 13–17), CL is considered incurable. Indeed, even though over 80% of dogs achieve complete remission upon induction chemotherapy, most of them relapse within 12 months (9, 10, 13–15). Moreover, relapsed patients frequently become refractory to therapy (18) and ultimately die of their disease, with a median overall survival time of only 10–14 months (13).

As in NHL and other canine cancers, increased risk, poor prognosis, and resistance to treatment in CL are very likely related to the genetic background of the host (19–26). Candidate gene, linkage and genome-wide association studies (GWASs) have revealed multiple genetic variants associated with NHL (25, 27–30). Specifically, genetic alterations associated with NHL include both single nucleotide variants and large rearrangements that place genes under the control of promoters and enhancers that are typically only active in the lymphocytes, leading to dysregulation of genes in pathways involving immune function, cell cycle, apoptosis, DNA repair, and carcinogen metabolism (31–33). Although genetic studies are still incipient for CL, evidence suggests that many of the same pathways dysregulated in NHL also exhibit perturbations in CL (31, 32). Nevertheless, how to use this information for patient stratification remains a matter of investigation (34).

Here we present the TiHoCL targeted sequencing panel, which comprises approximately 100 canine loci chosen using a combination of comparative genomics and network-based approaches. Our panel was designed starting from ~600 single nucleotide polymorphisms (SNPs) associated with lymphoma risk and prognosis in humans and only three in dogs, and was successfully technically and functionally assessed on 29 DNA samples extracted from whole blood of CL patients using the Ion Torrent™ Personal Genome Machine (PGM)™ technology. The TiHoCL targeted sequencing panel is a proof of principle to demonstrate how we can use knowledge from human research to develop clinical tools for other species. Facilitated by declining sequencing costs, the broad application of NGS panels like the TiHoCL targeted panel opens the door to the discovery of new drug targets and improved patient care.

## 2 Materials and methods

### 2.1 Identification of lymphoma-associated canine and human SNPs

First, we mined the Online Mendelian Inheritance in Man (OMIM) (35), the GWAS catalog (36), and PubMed for single nucleotide polymorphisms (SNPs). We accessed OMIM through their API; text searches for “lymphoma” were restricted to those containing allelic variants using the search field “av_exists.” The GWAS catalog was queried using the web interface; we searched for SNPs using the keyword “lymphoma” and required a *p*-value for the SNP-disease/trait association <1 × 10^−5^. Finally, we searched PubMed using the Entrez Programming Utilities (E-Utilities) (37) with the PubMed filters: “lymphoma[Title/Abstract] AND (“allelic variation” OR polymorphism) AND humans[MeSH]” (for human SNPs), and “lymphoma[Title/Abstract] AND (“allelic variation” OR polymorphism) AND dogs[Title/Abstract]” (for canine SNPs). The abstracts resulting from this query were searched for Reference SNP (rs) numbers. Next, we manually searched PubMed for articles published between the years of 2005 and 2015 with the keywords “lymphoma[MeSH]” and “genetic polymorphism[MeSH]” or “single nucleotide polymorphism[MeSH].” Any human or canine SNPs mentioned in the full text of these articles that were significantly associated (*p*-value < 10^−5^) with hematological malignancy risk, prognosis, outcome and resistance to treatment were selected as relevant.

We merged the SNPs resulting from all searches, removing duplicates and excluding those SNPs absent in dbSNP build 147 (38).

### 2.2 Identification of canine orthologous loci for human SNPs

To identify the canine orthologous locus of each human SNP we first retrieved the orthologous sequences of a 100-bp window centered at the human (GRCh38/hg38) SNP in the dog (CanFam3.1/canFam3), gorilla (gorGor4.1/gorGor4), mouse (GRCm38/mm10), and cat (ICGSCFelis_catus_8.0/felCat8) genomes with the LiftOver tool from the UCSC Genome Browser (39). SNPs in genomic windows for which no orthologous canine sequence was retrieved or for which the orthologous canine sequence exceeded 1,000-bp were excluded from further analyses. Finally, multiple sequence alignments were computed with T-Coffee v11.00.8cbe486 (40) to assess the evolutionary conservation of each SNP and flanking nucleotides. SNPs aligned to a gap in the canine genome were excluded from further analyses.

### 2.3 Genomic annotation of canine loci

Canine loci were annotated with the “annotatePeaks.pl” command from the HOMER v4.11.1 suite (41) using canFam3 as genome.

### 2.4 Gene annotation

Gene annotation was obtained from Ensembl [release 90, (42)] in GTF format.^1^

### 2.5 Identification of canine loci overlapping with UTRs

Gene annotation was filtered for features covering 50 bp or less. Bedtools intersect v2.27.1 (43) was used to identify overlaps with the remaining gene annotation features. Loci overlapping with annotated UTRs that did not overlap with coding exons in any transcript of the same gene or that overlapped with coding exons for less than two thirds of their length were reported as overlapping with UTRs.

### 2.6 Identification of canine loci for which the nearest upstream or downstream transcription start site is that of a gene annotated as a transcription factor

The nearest upstream and downstream TSS to each locus was identified based on the gene annotation with bedtools closest. The corresponding genes were classified according to the protein class that they encode (if any) using the online web server of the PANTHER Protein Classification System Database v11.0 (44).^2^

### 2.7 Identification of differentially expressed genes in CL patients

Differential expression analysis was performed on a total of 67 samples from three Gene Expression Omnibus [GEO, (45)] datasets: GSE112474, GSE30881, and GSE41917. GSE112474 is an RNA-seq dataset containing 12 B-cell, one T-cell, three intestinal lymphoma and four healthy lymph node samples. Intestinal lymphoma samples were considered as T-cell lymphoma samples (46). Differential expression analysis of RNA-seq read counts was performed using the R/Bioconductor package DESeq2 (47). B-cell and T-cell lymphoma libraries were compared to healthy lymph nodes separately. Genes with a *p*-value ≤ 1×10^−5^ were considered differentially expressed. GSE30881 and GSE41917 are gene expression microarray (Affymetrix Canine Genome 2.0) datasets. GSE30881 contains 23 diffuse large b-cell lymphoma lymph node samples and 10 healthy lymph node samples; GSE41917 includes seven B-cell, three T-cell lymphoma and four healthy lymph node samples. CEL files were processed and analyzed with the “simpleaffy” (48) and “affy” (49) R/Bioconductor packages. The robust multi-array average algorithm from the affy package was used for background correction and quantile normalization. Each disease sample group was independently compared to the healthy lymph node sample group. Variance estimators were computed with the empirical Bayes method in the “limma” (50) R/Bioconductor packages. Results were adjusted for multiple testing using the false discovery rate (FDR). Genes with a *log*_2_ fold-change above 2 and an FDR ≤ 0.05 were considered differentially expressed. The two microarray datasets were analyzed independently. Finally, the RNA-seq and microarray DEGs were pooled, removing duplicates. DEGs that could not be assigned to the chromosomes 1 to 38 or X were filtered out. A total 1,169 DEGs were identified in at least one of the comparisons.

### 2.8 Key regulators of gene expression in CL patients

The 1,000-bp sequences upstream of the TSS of the aforementioned 1,159 DEGs were searched for a maximum of 10 enriched motifs using MEME v4.11.2 (51) with parameters “-dna -nmotifs 5 -maxsize 500,000.” Identified motifs were compared to the JASPAR 2016 CORE vertebrate collection of motifs (52) with TOMTOM v4.11.2 (53) with parameters “-min-overlap 5 -dist pearson -evalue -thresh 10.0” to determine which transcription factor (TF) is likely to bind each motif. The analysis was performed separately for each sample group. A total of 21 TFs were identified as such.

### 2.9 Network analysis of DEGs in CL patients

The Search Tool for the Retrieval of Interacting Genes/Proteins [STRING, (54)] database v10.0 was used to investigate the interaction partners of the DEGs. Individual canine Ensembl gene identifiers were queried by means of the application programming interface (API). For example, the following URL was used to retrieve the interaction partners of “ENSCAFG00000000068”: http://version10.string-db.org/api/psi-mi-tab/interactionsList?identifiers=ENSCAFG00000000068&limit=500&required_score=500&species=9615 (last accessed July 20, 2023). The URL indicates the database, the access type (“api”), the output format (“PSI-MI TAB”), the list of requests (interaction partners for any of the query items), the gene of interest, the maximum number of network nodes (proteins) that are to be returned (“limit”), the threshold of significance to include an interaction (“required_score”), and the species (“9,615,” the taxonomy identifier for *Canis lupus familiaris*). After querying, we searched the output for interactions involving the Ensembl protein identifier(s) of the corresponding gene for which the score derived from curated data (“dscore”) was greater than 0.5.

### 2.10 Canine haplotype block map

Genotyping data for 60,968 SNPs in 600 dogs and 10 wolves (55–62) was kindly provided by Adam Boyko (Cornell University). The PLINK tool set v1.90 (63) was used to estimate the canine haplotype block structure. Because of the missing phenotype data, the option “no-phenoreq,” which removes the phenotype restriction, was added to the default settings. The coordinates of the haplotype blocks were converted from the assembly version CanFam2 to the assembly version CanFam3.1 using UCSC’s LiftOver tool.

### 2.11 Primer design and synthesis

Primers for generation of amplicons were designed based on sequences with a maximum length of 120 bp centered at the selected SNPs using the Ion AmpliSeq Designer v5.63 (Thermo Fisher Scientific, Waltham, MA, United States).

### 2.12 Canine lymphoma patient selection

Dogs referred to the Small Animal Clinic of the University of Veterinary Medicine Hannover for diagnostic investigation and treatment for CL between 2008 and 2014 were prospectively considered for enrollment in this study. Diagnosis was conducted based on cytological or histological evaluation of lymph nodes or extranodal lesions such as liver, spleen, and bone marrow. Patients underwent complete staging, consisting of history and physical examination, complete blood cell count, serum biochemistry profile, thoracic radiographs, abdominal ultrasound, cytological evaluation of liver and spleen regardless of their sonographic appearance, and bone marrow aspiration. Clinical staging was based on the World Health Organization (WHO) for canine lymphoma (64). Immunophenotype was determined by flow cytometry. The presence of the CD21 antigen and absence of CD3 antigen was considered diagnostic for B-cell lymphoma. All patients received standard treatment, namely CHOP followed by CCNU (lomustine), with or without L-asparaginase (see (65) for details). Inclusion criteria was a confirmed diagnosis of multicentric B-cell lymphoma. and absence of concomitant diseases that limited study compliance.

### 2.13 Clinical assessment

Response duration was assessed according to the Veterinary Cooperative Oncology Group (VCOG) consensus document (66). We reported response duration as progression-free survival (PFS), overall survival (OS). PFS was defined as the time from treatment initiation to first relapse; OS was defined as the time from treatment initiation to death from any cause. At the time of sequencing, 22 dogs had died from lymphoma-related causes, and seven were lost to follow-up.

### 2.14 Sample collection and DNA isolation

Before the first chemotherapeutic administration, blood samples of 29 dogs were collected for routine hematological and biochemical analyses. An aliquot of 200 μL retrieved from residual whole blood of each patient was preserved in EDTA and frozen in −80°C. DNA was extracted using the NucleoSpin Blood Kit (Macherey-Nagel) according to the manufacturer’s instructions. DNA quantification was carried out through Qubit 2.0 fluorometry (Thermo Fisher Scientific, Waltham, United States).

### 2.15 Library preparation and targeted NGS

From each sample, 10 ng of genomic DNA was used to prepare Ion Torrent sequencing libraries. Initial multiplex PCR was carried out at 2 min at 99°C, 18 cycles of 15 s at 99°C, 4 min at 60°C, and held at 10°C. Library quantification was performed via qPCR using Ion Library Quantitation Kit. Library nanosphere coupling and amplification was performed according to standard IonTorrent procedures using Ion OneTouch 2 and Ion OneTouch ES. Single-end sequencing was performed on an Ion Torrent^™^ Personal Genome Machine^™^ (PGM^™^) System using 318 v2 chips and 400 flows (Thermo Fisher Scientific, Waltham, United States).

### 2.16 Variant calling

The Torrent Suite software v5.4 (Thermo Fisher Scientific, Waltham, United States) was used to demultiplex reads, map the reads to the dog reference genome, and generate run metrics. Specifically, read mapping was performed against the dog reference genome [CanFam3.1^4^, (67)] using the Torrent Mapping Alignment Program (TMAP) with default cost values for mismatches (2) and indels (3) and minimum reference similarity (80%). Mapping quality (MAPQ) scores are reported in the Phred-scale.

Reads with mapping quality (MAPQ) below 90 were excluded using SAMtools view v1.10 (68). Duplicate marking was not deemed suitable for amplicon sequencing and thus omitted. Base calling was performed on the remaining reads with Freebayes v0.9.21.7 (69), GATK’s Haplotypecaller v4.1.7.0 (70), and BCFtools mpileup and call v1.9 (68). Each sample was processed separately. Reads used as input for Freebayes were realigned with ABRA2 v2.24 (71). Freebayes was run with default parameters; variants called across the entire genome were filtered for those within the amplicon regions with vcftools v0.1.16 (72). BCFtools mpileup was executed with parameters “-d 100,000 -L 100000” to ensure that all available reads were used for variant calling and that regions with high coverage were not skipped during indel calling; in addition, the “-r” parameter was used to restrict variant calling to amplicon regions. The output of mpileup served as input for BCFtools call, which was executed with the multiallelic and rare-variant calling model and parameter “-v” to output variant sites only (i.e., sites at which the sample had a non-reference allele). The reference genome dictionary and index required by GATK Haplotypecaller were generated with picard CreateSequenceDictionary v2.18.29 (73) and GATK IndexFeatureFile, respectively. GATK BaseRecalibrator and ApplyBQSR were used for base quality recalibration; all variants in the Ensembl Variation database^5^ were used with the parameter “--known-sites.” Finally, GATK Haplotypecaller was run restricting the analysis to the panel amplicons (with the “--intervals” parameter) and with parameter “--max-reads-per-alignment-start” set to 0 to disable read downsampling. The variants called with each of the three variant callers were then filtered with BCFtools view so that only variants with a quality score ≥20 were kept for further analyses and normalized with BCFtools norm to left-align and normalize indels. Finally, BCFtools isec was used with parameters “--collapse both” (to collapse SNPs and indels) and “--nfiles +1”to identify the variants called by 1, 2, and 3 variant callers in each of the samples. Variants called by all three variant callers in at least one sample are subsequently referred to as “pooled.” Only sites that were not called variants by any of the three variant callers were considered homozygous for the reference allele.

Pooled variants were annotated with the Variant Effector Predictor (VEP) v109.3 (72, 74).

### 2.17 Survival analyses

Survival analysis was performed for PFS and OS using the R packages “survminer” v0.4.9 (75), “survival” v3.5–5 (76), and “MASS” v7.3–53 (77). For OS, the seven patients lost to follow-up at the time of sequencing were censored at the latest date of available records.

Univariable Cox regression (78) was performed using the cophx() function of the “survival” package to analyze associations with the covariates “WHO clinical stage,” “sex,” “neuter status,” “chemotherapeutic protocol,” and “age at diagnosis.” All variables were treated as categorical; “age at diagnosis” was encoded as 1 for “adult” (2–7 years) and 0 for “senior” (8–11 years).

Pooled variants homozygous or heterozygous for the alternate allele in 11 to 18 dogs and homozygous for the reference allele in all other dogs of the study cohort were subjected to survival analysis following the approach from Collett (79). Briefly, we first performed a univariable Cox regression for each variant using the cophx() function of the “survival” package. Next, we included variants with a *p*-value ≤ 0.5 in a multivariable Cox regression model, and conducted stepwise backward selection with the stepAIC() function of the “MASS” package to select a combination of variants with lowest Akaike Information Criterion (AIC) value. All variants with a *p*-value ≤ 0.1 in the resulting model were used for multivariable Cox regression with stepwise forward selection of any variables with a *p*-value ≤ 0.5 in the univariate Cox regression. Variants with a *p*-value ≤ 0.1 in the resulting model were used for stepwise bidirectional –forward and backward– regression. Variants with a *p*-value ≤ 0.05 in the final model were considered significant.

## 3 Results

### 3.1 Database mining unveiled 482 putative lymphoma-associated canine loci

**Figure 1:**
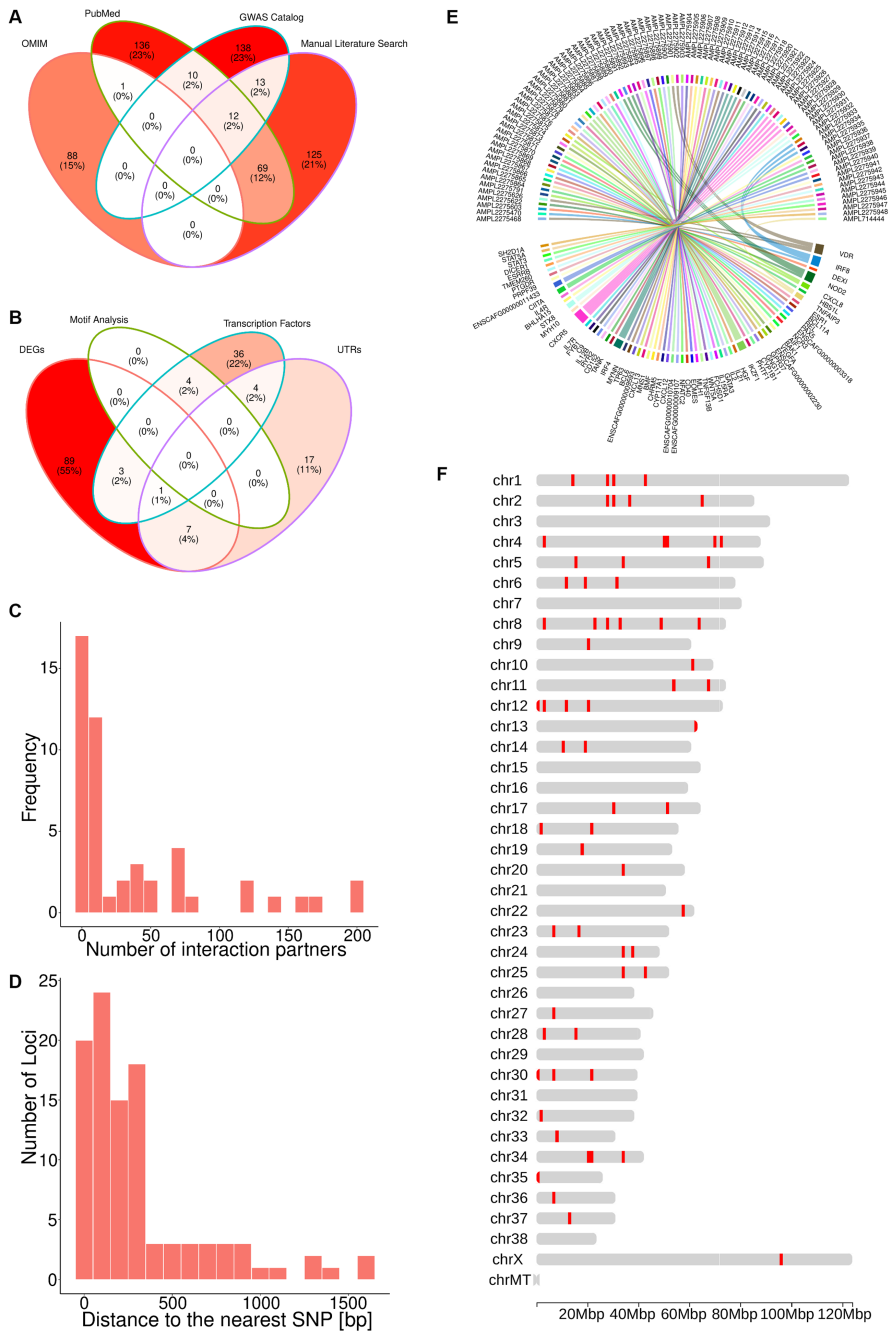
The TiHoCL targeted sequencing panel relies on SNPs associated with NHL to detect variants in CL. **(A)** Venn diagram showing the sources of the 592 human lymphoma-associated SNPs; 89 SNPs were retrieved from the OMIM, 173 from the GWAS Catalog, 228 from PubMed, and 219 through manual literature curation. **(B)** Venn diagram displaying how the SNPs from **(A)** were selected based on the different filtering strategies. The brightness of the filling color in **(A,B)** corresponds to the number of SNPs. **(C)** Number of interaction partners for the DEGs. **(D)** Distance between selected 98 potential lymphoma-associated loci and the nearest known SNPs in the canine genome, which was also a filtering criteria for the SNPs. **(E)** Circos plot showing the nearest gene for each target. **(F)** Distribution of the 93 targets across the dog genome.

To identify putative lymphoma-associated loci in the canine genome, we first mined the Online Mendelian Inheritance in Man (OMIM) database (35), the GWAS catalog (36), and PubMed titles and articles for human or canine SNPs (Methods). This Initial search yielded 89, 173, and 228 human SNPs, respectively. Furthermore, we manually reviewed all full text articles about NHL and CL indexed in PubMed, identifying 219 human and three canine SNPs (Methods). In total, this accounted for 592 non-redundant human (Figure 1A) and three canine SNPs. These three canine SNPs were the only SNPs predisposing for canine B-cell lymphoma that could be identified by a GWAS involving 41 cases and 172 controls (80).

To assess the evolutionary conservation of the 592 human SNPs and identify their orthologous canine loci, we computed multiple sequence alignments between 100 bp-long sequences centered at each human SNP and their orthologous sequences in the dog and three additional species’ genomes (Methods). Thereby, we found canine orthologs for 524 (89%) human sequences, but discarded one that diverged considerably in length (Methods) and one on pseudo-chromosome “chrUn” (contigs that could not be assigned to any chromosome). From the remaining 522 canine sequences, 43 contained insertions or deletions at the site orthologous to the SNP in the human sequence, and thus were not considered evolutionary conserved. Excluding these 43 sequences yielded 479 putative lymphoma associated canine loci.

Overall, taking into account the three canine SNPs reported in the literature, we discovered 482 putative lymphoma associated canine loci. Of these, 420 (87%) were non-protein-coding.

### 3.2 Functional genomics and network approaches identified high-priority loci for CL patient management

Since the aim was to develop a fast and relatively inexpensive SNP genotyping panel for diagnosis and monitoring of CL patients, we applied a combination of functional genomics and network approaches to single out approximately 100 most promising putative lymphoma-associated canine loci out of the aforementioned 482.

First, we selected the 29 loci located within untranslated regions (UTRs) and 48 loci for which the nearest upstream or downstream TSS was that of a gene annotated as a TF (Methods). Variants in UTRs may alter transcription binding sites, splicing sites and polyadenylation of mRNAs (80–83). Similarly, variants within or in the neighborhood of TF genes may lead to aberrant TF expression and, in turn, gene dysregulation of almost all known cellular processes related to tumorigenesis (84), which could explain the widespread gene expression dysregulation observed in lymphoma (85, 86).

Next, we examined the loci in the neighborhood of genes found to be differentially expressed in lymphoma in several transcriptomic studies (Methods). We found four loci located in the Vitamin D Receptor gene, which encodes a putative key regulator of gene expression in lymphoma. Because genetic variation in this gene has been associated with lymphomagenesis (87–94), these four loci were also selected for the panel. Further, the nearest TSS to 100 of the loci corresponded to a DEG. Eleven of these loci were also in the neighborhood of a TF gene or within a UTR and, hence, had already been selected for the panel (Figure 1B). From the remaining loci, we selected 29 near protein-coding genes that possessed more than 10 interaction partners (Methods). Genes with a large number of interaction partners are referred to as network “hubs” (Figure 1C), and hubs are typically deemed important, because their perturbations can have major consequences for the regulatory networks to which they contribute (95).

In total, the aforementioned approaches yielded 101 loci in the canine genome representing the orthologous sites of human SNPs. Importantly, these loci are not necessarily polymorphic in the canine genome. In order to associate them with known canine SNPs, we constructed and analyzed a canine haplotype block map (Methods). We observed that the median block size over all individuals was 5,725 bp and that all loci of interest were located at a distance smaller than the median haplotype block size from a known SNP in the canine genome, with a median distance of 195 bp and a maximum of 1,616 bp (Figure 1D). This implies that the loci of interest and their nearest SNPs are very likely to be in linkage disequilibrium, and hence, we chose the latter to represent the former in our panel. Since some of the loci were located in the proximity of the same SNP, the total number of SNPs associated with the loci of interest was 100.

### 3.3 The TiHoCL sequencing panel targets 93 SNPs in the canine genome

The 100 canine SNPs derived from the multi-step genomic analyses presented above together with the three SNPs that had been reported in the literature to be associated with CL were used for designing the TiHoCL targeted sequencing panel. Using the Ion Ampliseq Designer (Methods) we were able to design primers for 93 of the 103 SNPs (Figures 1E,F). The resulting custom, single pool, multiplexed, PCR-based, NGS library panel comprised 93 target amplicons with an insert size between 220 and 335 bp in length (median = 324 bp), each covering one of the aforementioned 93 SNPs, and had a total size of ~30 kb (Supplementary Table S1). We further refer to the target amplicons as *targets*.

### 3.4 TiHoCL targeted sequencing panel identifies variants in CL samples

We technically validated the TiHoCL targeted sequencing panel using DNA extracted from a cohort of 29 dogs presenting B-cell multicentric lymphoma (Supplementary Table S2). The most encountered breeds were mixed-breed dogs (6/29, 21%), Bernese Mountain Dog (5/29, 17%), Golden Retriever (2/29, 7%), and Rottweiler (2/29, 7%), but other 14 additional breeds were found (14/29, 48%). There were 18 males and 11 female dogs; half of them were neutered, and half were intact. The age at diagnosis ranged from two to 11 years (mean [SD]: 6.45 [2.21] years). Weight was recorded for 25 dogs; they weighed between 6.8 and 51 kg. According to the WHO clinical stage classification, 2 dogs had stage III disease, 16 dogs stage IV disease, and 11 dogs stage V disease. Six dogs were treated with a 12-week CHOP protocol followed by three doses of CCNU (lomustine), while 23 dogs received the same protocol with the addition of L-asparaginase in the first week. A group of five patients relapsed before the induction phase was finished, and all 29 patients relapsed before the end of the study period (24 months). Univariable Cox regression revealed no association between WHO clinical stage classification, sex, neuter status, treatment protocol, and age at diagnosis and PFS or OS (Supplementary Table S3).

**Figure 2:**
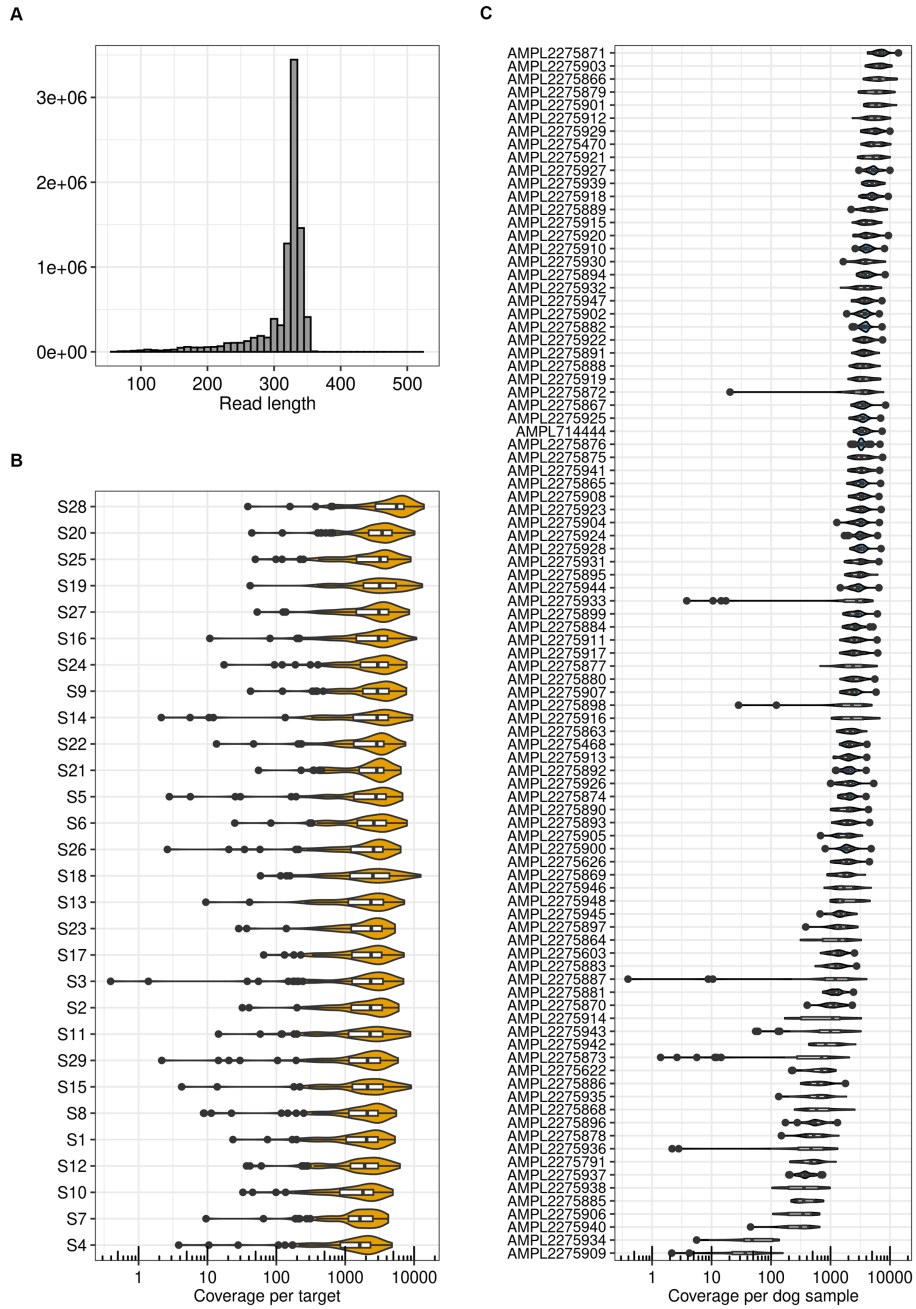
TiHoCL targeted sequencing panel achieved good run metrics. **(A)** Distribution of read lengths obtained after mapping quality (MAPQ) filtering with the panel on 29 dog samples. **(B)** Target coverage per sample. The Median coverage was greater than 500X for all samples. **(C)** Coverage of all samples per target. For seven targets the median coverage was smaller than 500X, two of them being outliers with a median coverage smaller than 100X.

Sequencing run metrics showed good data quality for all samples (Supplementary Table S4). Uniformity of base coverage across samples ranged from 89 to 96%. After quality filtering, the average read length per sample ranged between 306 and 318 bp with an average of 312 bp (Figure 2A). Depending on the sample, the number of reads mapped to targets ranged from 188,712 to 582,628. This corresponds to a percentage of reads on target between 98.50 and 99.99%, confirming adequate target design, library preparation, and sequencing. The median target coverage exceeded 1,000X, with an average over all samples of 2,612X (Figure 2B). Similarly, the median sample coverage was greater than 500X, with only nine exceptions, with an average over all targets of 2,641X (Figure 2C). This is in line with the minimum coverage generally recommended for clinical oncology panels (96). In addition, 77 to 88% of the bases in the targets indicated no strand bias. Because of the satisfying run metrics we continued with functional validation.

To assess the level of polymorphism of the targets we performed variant calling (Methods). To obtain a reliable list of potential variants, we used three different variant callers: Freebayes, GATK Haplotypecaller, and BCFtools mpileup and call, and solely considered variants called by all three variant callers for downstream analysis. The three variant callers were chosen because they are partly based on different approaches and are broadly used in the field (97–100).

**Figure 3:**
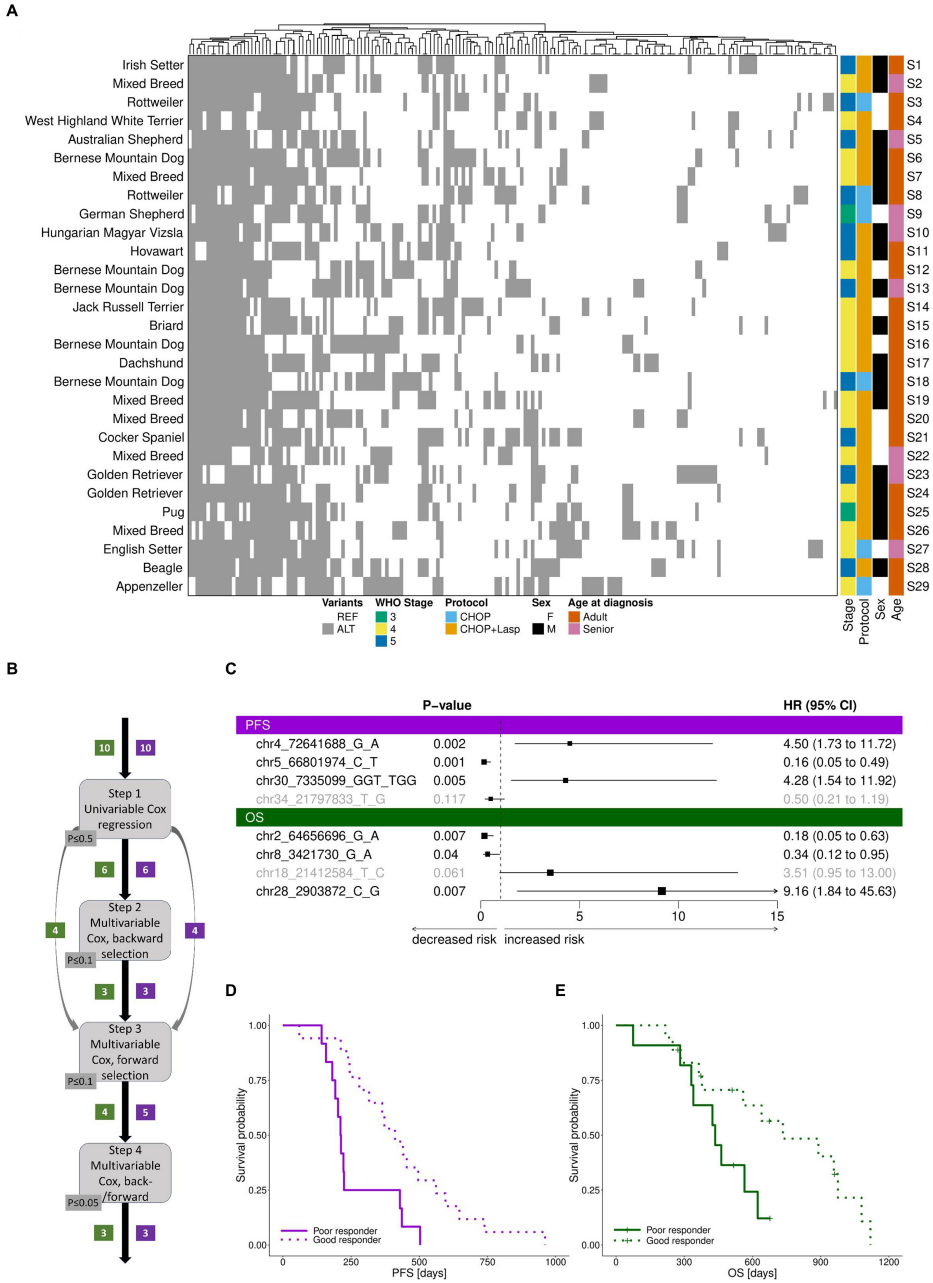
TiHoCL targeted sequencing panel enabled detection of natural genetic variation in the study cohort and of variants associated with CL relapse and survival. **(A)** Heatmap of polymorphic “pooled” variants across 29 dog samples. Variants are indicated with respect to the reference genome. REF: reference allele; ALT: alternative allele. No association was observed between WHO clinical stage classification, sex, neuter status, treatment protocol, and age at diagnosis and PFS or OS using univariate Cox regression modeling. Heatmap columns were clustered hierarchically using complete linkage, based on their euclidean distances. Heatmap rows were sorted according to sample number. **(B)** Graphical workflow of the approach applied to identify genetic variants associated with PFS and OS. The numbers in boxes indicate the number of genetic variants selected in the corresponding step for PFS (violet) and OS (green). **(C)** Forest plot of cox regression hazard ratios for analysis of PFS and OS. A significant *p*-value and a hazard ratio smaller than 1 indicate a strong relationship between the variant and a decreased risk of death (OS)/relapse (PFS); a significant *p*-value and a hazard ratio greater than 1 indicate a strong relationship between the variant and an increased risk of death (OS)/relapse (PFS). Non-significant variants in the models are displayed in gray. The variant chr30:7335189G > C, which is placed on the same target and features the same pattern as chr30:7335099GGT > TGG, was removed prior to the analysis, but would also have a positive hazard ratio for PFS. Similarly, the variant chr2:64656755G > A, which is placed on the same target and features the same pattern as chr2:64656696G > A, was removed prior to the analysis, but would also have a negative hazard ratio for OS. **(D,E)** Survival based on samples separated by the median risk scores derived from the final cox models for PFS **(D)** and **(E)** OS.

Compared to the reference genome, we detected a total of 188 “pooled” (heterozygous or homozygous) variants (Figure 3A, Supplementary Table S5), with each sample exhibiting between 56 and 96 variants (Methods). For 10 of the variants, all 29 samples were homozygous for the same allele (but polymorphic relative to the reference genome sequence). Of the 188 pooled variants, the majority (81%) were SNPs, followed by indels (19%). Consistent with the panel design, the 87% were non-protein coding. From the original 93 SNPs used to design the panel, 70 were also polymorphic relative to the reference genome sequence in the samples. Each target exhibited an average of 0.7 variants per sample.

Of the pooled variants, 12 were seen in 11–19 dogs, and considered polymorphic in the cohort. We checked this list for variants showing the same ALT/REF pattern and removed the duplicated ones for the next step which left 10 variants. These variants were then subjected to multistep survival analysis using Cox proportional hazards regression (Figure 3B). The Cox (proportional hazards or PH) model describes the relation between the (cumulative) incidence of an event of interest (in our case, death for OS and relapse for PFS) and a set of covariates (in our case, genetic variants), and is a commonly used approach for analyzing survival time data in medical research. Using univariate Cox regression analysis, we first identified 6 variants weakly associated with overall survival (OS). Then, we applied multivariable Cox regression analysis to establish a prognostic model. The final model included three variants (Figure 3C). Among them, one was associated with increased risk of death and two were associated with decreased risk of death. For PFS, the same procedure resulted in a model including three variants (Figure 3C), of which two were associated with increased risk of relapse and one was associated with decreased risk of relapse. The final models clearly distinguished patients into poor (median OS: 436 days, median PFS: 212 days) and good responders (median OS: 600 days, median PFS: 411; Figures 3D,E).

To summarize, the TiHoCL targeted sequencing panel showed good run metrics and allowed us to characterize known and novel genetic variation in the cohort. Furthermore, we identified eight variants associated with a decreased and increased risk of relapse or death.

### 3.5 TiHoCL targeted sequencing panel is labor- and cost-saving

The total costs for sequencing on an Ion Torrent PGM platform, including primer pool, library preparation, emulsion PCR and massively parallel sequencing, are approximately 220 € per sample using the maximum capacity of a 318 v2 chip with 16 samples. Initially, the 318 v2 Chip allows 5×10^6^ reads, thus considering the uniform target amplification kinetics of the TiHoCL Panel, 100 samples could be sequenced with coverage of 500X. However, as sequencing efficiency is strongly dependent on chip-loading, the sequencing of 16 to 29 samples per chip will be delivering easily 1,000X coverage of all analyzed bases for less than 250€ per sample in 2 to 5 h.

## 4 Discussion

The widespread use of targeted sequencing panels in human oncology (101) anticipates new directions for veterinary science, wherein similar panels have the potential to revolutionize our understanding and management of cancer in animals. Recent technological developments illustrate the significance and viability of this. For example, Sirivisoot and colleagues (102) used mass spectrometry to assess variation in 41 loci previously reported to drive lymphomagenesis and associated with genes producing targetable proteins, and found that their panel provides useful prognostic information when screening a cohort of 60 dogs with diffuse large B-cell lymphoma. To move the research to the next level, we demonstrate how functional genomics and network approaches can be combined to select SNPs mined from the literature with the aim of designing a targeted panel for CL. This panel was evaluated in a cohort of 29 CL patients, achieving satisfactory sequencing metrics, capturing natural polymorphisms in the canine population, and exhibiting a promising potential to stratify CL patients.

Although our knowledge about the molecular mechanisms underlying CL is limited, CL resembles the much better researched human disease NHL in many regards, and this can be used to design tools that can help guide treatment decisions in CL patients.

Our panel design primarily relied on knowledge from the literature and the OMIM and GWAS catalog databases. Automatic text mining resulted in 467 SNPs. Manual inspection of the full text of selected PubMed articles produced 125 additional SNPs. In this manner, we identified a total of 592 human and three canine SNPs. These SNPs were then filtered for the most promising candidates using a combination of functional genomics and network biology approaches to build a CL panel including approximately 100 loci. Including a relatively small number of loci in the panel design enabled us to subject samples to deep sequencing—which is considered superior over technologies such as whole-genome sequencing, whole-exome sequencing, and PCR when analyzing multiple mutational spots in parallel (103, 104)—at an affordable cost. On these grounds we specifically prioritized loci within UTRs, near TFs, or near genes that are differentially expressed in CL patients and code for proteins that act as “hubs” in relevant regulatory networks. The TiHoCL panel was technically and preliminary functionally assessed on a cohort of 29 CL patients. Most (90%) amplicons performed well, reaching a coverage of 500X. Furthermore, the panel detected 56 to 96 variants in each CL patient, successfully capturing some of the natural genetic variation within the cohort and paving the way for personalized CL therapy. Highly encouraging, a stringent multistep approach to variable selection and survival analysis on a set of 12 variants detected in more than 10 patients identified four variants associated with decreased or increased risk of death, and another four, of relapse. These variants merit further investigation for validation as prognostic biomarkers.

Two main limitations should be acknowledged. First, our panel was based on a relatively small number of SNPs extracted from the literature and specialized databases, which only partially represent the scientific knowledge about CL. In particular, we restricted ourselves to two manually-curated, high-quality databases to increase our chances of including high-confident SNPs in the panel design, but those are not the only –or the most comprehensive– databases that can be used as source of disease-associated SNPs. For future panel designs it could be worth considering databases like ClinVar (105), which is focused on disease associated SNPs, COSMIC (105, 106), which is focused on SNPs for cancer research and diagnostics, or dbVar (107), a database for human structural variation to cover also larger rearrangements. In addition, our search did not distinguish between germline and somatic variation, and thus, the SNPs finally included in the panel were not optimized for patient stratification and management, which is the ultimate goal of the TiHoCL panel. Indeed, such restriction appeared excessive in light of the scarce genetic knowledge available for CL. Second, functional assessment of the panel was performed on a cohort of only 29 dogs, which may not represent the full spectrum of CL genetic subtypes and canine breeds. Although our results provide preliminary evidence about the functional capabilities of the TiHoCL panel, we acknowledge that the variants identified as being associated with risk of relapse or death warrant further examination. The decision on the cohort size and diversity was made considering the exploratory nature of our study and the constraints imposed by cost, time and case incidence. While focusing on specific, lymphoma-prone breeds would help reduce genetic background heterogeneity and population structure, leading to more reliable results, this approach could miss important genetic interactions, and would have limited utility for mixed-breed dogs. Breed diversity is also supported by a study of Elvers and colleagues (108) indicating that breeds predisposed to B-cell lymphoma have commonly mutated genes and pathways. A larger cohort would increase the statistical power of the study, and improve the reliability and robustness of our findings. In summary, knowledge transfer to clinical practice would entail leveraging the most current information for panel design and an extensive functional validation of the thereby enhanced TiHoCL panel on a larger and more diverse cohort.

Our design and assessment framework is a proof of principle, representing a foundation upon which other targeted sequencing panels could be built. The version of the TiHoCL targeted sequencing panel presented here can be seen as the first stage in an iterative development model. Our framework streamlines the establishment of refined and updated versions of the TiHoCL panel. As genomic data for lymphoma accumulates, new loci could be periodically incorporated into the panel, especially those arising from progressively more accessible whole-genome sequencing studies, while systematically removing loci with limited potential. With each iteration, the panel’s performance is set to increase, enabling novel opportunities for enhancing CL patient management. Our frameworks flexibility ensures its enduring relevance in the constantly evolving landscape of cancer research and propels the field of precision veterinary medicine to new heights.

Because human diseases are generally more thoroughly researched and documented, most genetic databases, including OMIM and the GWAS catalog, are limited to human variants. Moreover, most of the SNPs obtained from the literature were human SNPs. Therefore, the TiHoCL targeted sequencing panel for CL was primarily built based on knowledge acquired for a different, but related disease, namely NHL. This concept is transferable to other cancer types or diseases that share features with diseases in other species, like sarcomas in dogs or urothelial neoplasms in sea lions (109). In contrast to the situation in human medicine, advanced molecular analysis tools for veterinary patients are scarce, limiting clinical options. The reasons for this are manyfold, but economic factors should not be underestimated. Thus, many more resources are invested in human than in dog cancer research. In addition, while many costs in human medicine are often covered by national health care systems, veterinarian costs are normally covered by the animal’s owner. Of course, our design framework could also be beneficial for human medicine and have an impact on translational medicine. Clinical trials in dogs with spontaneous cancers must be conducted ethically and in compliance with relevant animal welfare regulations, but offer several advantages compared to studies in humans, including reduced liability and faster approval timelines. CL is a unique spontaneous model for NHL, and therefore, can be used to understand NHL progression and drug resistance processes, as well as to develop new treatments (9, 10, 14). Furthermore, while certain diseases might be rare in humans and hence, difficult to study, they could occur frequently in dogs, providing a unique perspective for research.

The broad application of panels similar to our TiHoCL targeted sequencing panel for CL not only has the potential of improving the health and welfare of all species suffering from cancer, but also contribute to our understanding of the underlying pathology.

## Data availability statement

The datasets presented in this study can be found in online repositories. The names of the repository/repositories and accession number(s) can be found at: https://www.ncbi.nlm.nih.gov/, PRJNA1017182.

## Ethics statement

The animal studies were approved by the Small Animal Clinic of the University of Veterinary Medicine Hannover, Foundation (Hanover, Germany) in accordance with the German Animal Welfare Guidelines, approved by the Ethics Committee of the State of Lower Saxony, Germany (No. 14/1700). None of the dogs were euthanized due to reasons of sample collection. The studies were conducted in accordance with the local legislation and institutional requirements. Written informed consent was obtained from the owners for the participation of their animals in this study.

## Author contributions

SF-S: Writing – original draft, Data curation, Formal Analysis, Methodology, Software, Visualization, Writing – review & editing. CI: Data curation, Writing – original draft, Writing – review & editing. DS: Data curation, Formal Analysis, Software, Writing – review & editing. NE: Data curation, Writing – review & editing. JG-S: Data curation, Writing – review & editing. WL: Data curation, Writing – review & editing. SK: Investigation, Writing – review & editing. CJ: Funding acquisition, Project administration, Writing – review & editing. IN: Funding acquisition, Methodology, Project administration, Writing – review & editing. LT: Conceptualization, Supervision, Writing – original draft, Writing – review & editing. HM: Conceptualization, Supervision, Writing – original draft, Writing – review & editing.

## Footnotes

^1^https://ftp.ensembl.org/pub/release-90/gtf/canis_familiaris/Canis_familiaris.CanFam3.1.90.gtf.gz, last accessed July 20, 2023.

^2^http://pantherdb.org/, last accessed July 20, 2023.

^4^ftp://ftp.ensembl.org/pub/release-90/fasta/canis_familiaris/dna/, last accessed on September 2017.

^5^https://ftp.ensembl.org/pub/release-100/variation/vcf/canis_lupus_familiaris/canis_lupus_familiaris.vcf.gz, last accessed on October 25, 2022.
